# Antibacterial effects assessment on some livestock pathogens, thermal stability and proposing a probable reason for different levels of activity of thanatin

**DOI:** 10.1038/s41598-021-90313-4

**Published:** 2021-05-25

**Authors:** Ali Javadmanesh, Elyas Mohammadi, Zahra Mousavi, Marjan Azghandi, Abass Tanhaiean

**Affiliations:** 1 Department of Animal Science, Faculty of Agriculture, Ferdowsi University of Mashhad, Mashhad, Iran 9177948974; 2 Stem Cell Biology and Regenerative Medicine Research Group, Research Institute of Biotechnology, Ferdowsi University of Mashhad, Mashhad, Iran; 3 Faculty of Pharmacy and 3P Medicine Laboratory, International Research Agendas Programme, Medical University of Gdańsk, Gdańsk, Poland; 4 Department of Plant Breeding, Faculty of Agriculture, Shahrood University of Technology, Shahrood, Iran

**Keywords:** Peptides, Structural biology, Biochemistry, Microbiology

## Abstract

There is a continuing need to prevent the increasing use of common antibiotic and find the replacement to combat the drug/antibiotic resistant bacteria such as antimicrobial peptides (AMPs) such as thanatin peptide. In this study, recombinant thanatin peptide was expressed in the HEK293 cell line. Then the antimicrobial properties of this peptide on some poultry and farm animal’s pathogen strains were assessed. The thermal-stability of thanatin was predicted in various temperatures through in silico analysis. Afterwards, according to Minimum Inhibitory Concentration (MIC) results, *Escherichia coli* and *Pseudomonas aeruginosa* were chosen to test the hypothesis of LptA/LptD–thanatin interaction, computationally. Relative amino acid sequences and crystallography structures were retrieved and missed tertiary structures were predicted. The interaction of thanatin with LptA and LptD of *Escherichia coli* and *Pseudomonas aeruginosa* were analyzed subsequently. The antibacterial activity of thanatin peptide was evaluated between 6.25 and 100 μg/mL using minimum inhibitory concentration. Also, the amounts of minimum bactericidal concentrations (MBC) were between 12.5 and 200 μg/mL. The bioinformatics analysis followed by the in vitro assessment, demonstrated that thanatin would be thermally stable in the body temperature of poultry and farm animals. Thanatin could penetrate to the outer membrane domain of LptD in *Escherichia coli* and it could block the transition path of this protein while the entrance of LptD in *Pseudomonas aeruginosa* was blocked for thanatin by extra residues in comparison with *Escherichia coli* LptD. In addition, the quality of interaction, with regard to the number and distance of interactions which leads to higher binding energy for thanatin and LptD of *Escherichia coli* was much better than *Pseudomonas aeruginosa.* But the site and quality of interaction for thanatin and LptA was almost the same for *Escherichia coli* and *Pseudomonas aeruginosa.* Accordingly, thanatin can prevent the assembly of LptA periplasmic bridge in both pathogens. The antibacterial and thermal stability of the thanatin peptide suggested that thanatin peptide might serve as a natural alternative instead of common antibiotics in the veterinary medicine. The outcome of this in silico study supports the MIC results. Therefore, a probable reason for different level of activity of thanatin against *Escherichia coli* and *Pseudomonas aeruginosa* might be the quality of LptA/LptD–thanatin interaction.

## Introduction

The spread of antibiotic-resistant bacteria poses a major threat to the public health crisis. Extensive antibiotic uses in animal production for preventing and treating disease are between many factors that might contribute to antibiotic-resistance which leads to economic losses^1, 2^. In livestock industries, the bacterial strains that lead to antibiotic-resistant are *Escherichia coli, Salmonella* spp., and *Pseudomonas* spp.^3^. Additionally, there are anxiety about the antibiotic residuals in meat, milk, and eggs, and after that transmission to humans through the food that endangers human safety^2^.

Globally, there is a continuing need to prevent the increasing use of common antibiotics and find the replacement to combat the growing bacteria such as antimicrobial peptides (AMPs)^4^. There is a significantly increasing interest in identifying new classes of AMPs. Thanatin has received great attention along with activity against Gram-negative and positive bacteria^5, 6^, as well as drug-resistant bacteria^7, 8^. Additionally, thanatin peptide might improve the antibacterial activity of some antibiotics such as chloramphenicol, norfloxacin on multidrug-resistant *Enterobacter aerogenes* through the energy-dependent efflux mechanism, and persuaded a remarkable improvement of antibiotic susceptibility contrast sensitive cells^9^. Unfortunately, some reasons exist for preventing the use of some antimicrobial peptides such as hemolytic toxicity^10^, as well as the emerging of drug resistance^11^. Several studies demonstrated that thanatin was not toxic on some human cell lines even at very high concentrations which is considered an appropriate feature in utilizing this peptide in the treatment section ^10, 12^. Unlike pore-forming peptides, the primary mechanism of thanatin was through agglutination and clumping of cells that culminate reducing of releasing toxic substances by bacteria^13, 14^.

Recently, another mode of action for thanatin is proved. Gram-negative bacteria are protected against cytotoxic molecules by the asymmetric outer membrane (OM). Integral OM proteins play a vital role in the biogenesis of the OM. Besides, these proteins control the uptake and export of signaling molecules and nutrients across the OM. The incorporation of lipopolysaccharide (LPS) into the OM of Gram-negative bacteria is accomplished by seven proteins (LptA to LptG). The mentioned proteins form a macromolecular complex that spans the entire envelope^14, 15^. The LptA is a periplasmic protein that forms a bridge spanning the periplasm. The LPS molecules are pushed across this bridge^16, 17^ to the LptDE complex embedded in the OM^18, 19^.

It has been shown that the thanatin targets both LptA and LptD in *Escherichia coli.* Antimicrobial activity of thanatin was reported against *Escherichia coli* and *Pseudomonas aeruginosa* which shows the lower activity of this peptide against *Pseudomonas aeruginosa*. The NMR complex of thanatin and LptA of *Escherichia coli* (PDB entry: 6GD5) is available but there is no clue about these interactions for LptA of other pathogens. Besides, the information about LptD-thanatin interactions would be desirable due to the lack of considerable information on this case.

In this study, we used the recombinant thanatin peptide from our previous study^12^ to assess the minimum inhibitory concentration and the minimum bactericidal concentration on some farm animal and poultry bacterial pathogens. Furthermore, the thermal-stability of thanatin was investigated by both in silico and a culture-based method. We have evaluated the interaction of thanatin with LptA and LptD proteins of *Escherichia coli* and *Pseudomonas aeruginosa* to see if the likely reason of different level of activity of thanatin against these two pathogens, is the mentioned interaction or not.

## Results

### MIC and MBC assays of the thanatin peptide

The concentration of thanatin was estimated around 400 µg/mL. The results of MIC and MBC assay against typical Gram-positive and Gram-negative bacteria are summarized in Table 1. According to the results, the lowest and the highest values of MIC were observed against *Escherichia coli* O157:H7*, Salmonella Enteritidis* from cattle mastitis (6.25 μg/mL) and *Salmonella Enteritidis* from poultry diarrhea (100 μg/mL), respectively. Moreover, the lowest and the highest values of MBC were observed in *Escherichia coli* O157:H7 (12.5 μg/mL) and *Salmonella Enteritidis* from poultry diarrhea*, Salmonella Typhimurium* (200 μg/mL), respectively.

**Table 1:** Comparative MIC and MBC of the thanatin in various Gram-positive and negative bacteria.

Strains	MIC (µg/mL)	MBC (µg/mL)
**Poultry bacteria**		
*Staphylococcus aureus*^b^	25	50
*Escherichia coli*^a^	25	100
*Escherichia coli k99*^a^	12.5	25
*Klebsiella pneumoniae*^b^	> 200	> 200
*Salmonella enteritidis*^b^	25	50
*Pseudomonas aeruginosa*^b^	> 200	> 200
*Salmonella enteritidis*^a^	100	> 200
*Salmonella typhimurium*^a^	50	200
**Farm animal bacteria**		
*Klebsiella pneumoniae*^c^	> 200	> 200
*Salmonella enteritidis*^c^	6.25	25
*Staphylococcus aureus*^c^	12.5	25
*Escherichia coli* O157:H7^d^	6.25	12.5

Data are collected as MICs according to the CLSI (www: clsi.org).

All bacteria were obtained from Mashhad School of Veterinary Medicine Collection, Ferdowsi University of Mashhad, Iran.

*ND* not determined.

^a^From poultry diarrhea.

^b^From poultry intestine infection.

^c^From cattle mastitis.

^d^From calf diarrhea.

In this study thanatin did not show any activity against *Klebsiella pneumoniae* (from poultry intestine infection), *Klebsiella pneumoniae* (from cattle mastitis), and *pseudomonas aeruginosa* (from poultry intestine infection). As a result, the information demonstrated this peptide had beneficial antimicrobial activity against some bacterial species isolated from poultry and farm animal infections.

### In silico thermal-stability

**Figure 1:**
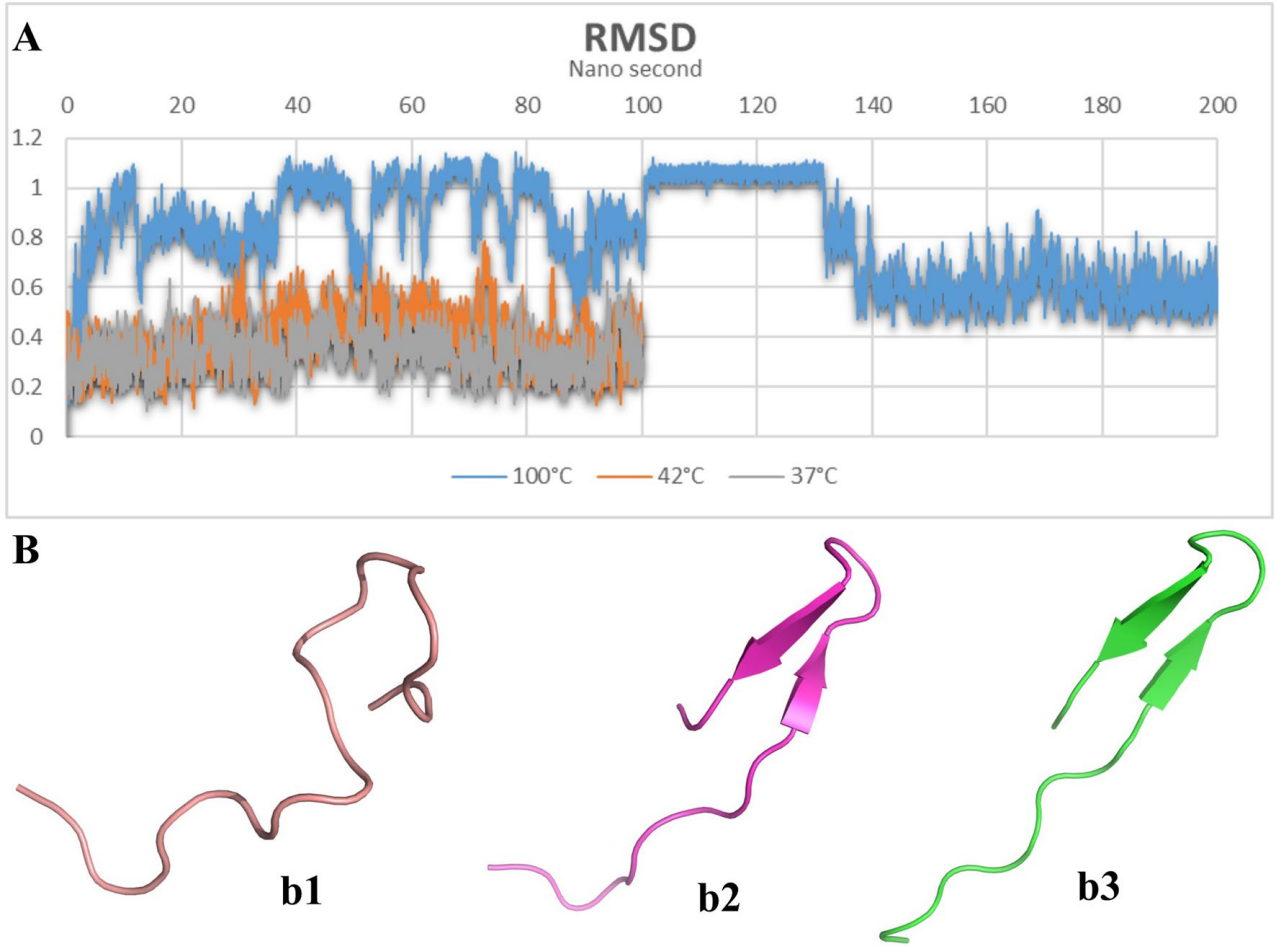
(**A**) The root-mean-square deviation (RMSD) of thanatin α-carbons (nm) versus time (ns). (**B**) The tertiary structure of thanatin in (b1) water boiling temperature at sea level, (b2) avian normal body temperature, (b2) dairy cattle normal body temperature.

The root-mean-square deviation (RMSD) results (Fig. 1A) for the prediction of thermal-stability of thanatin in normal body temperature of dairy cattle (312 K), avian (315 K), and water boiling temperature at sea level revealed that this protein is thermo-stable in all three conditions. Thanatin was stable from the beginning to the end of simulation in normal dairy cattle and avian body temperatures but the fluctuations of RMSD for thanatin in water boiling temperature depict that this peptide was not stable during the first 150 ns. After an almost steady line between 100 and 150 ns which shows a temporary stability, the RMSD falls dramatically and the value of RMSD remained steady until the end of simulations with an almost 0.1 nm deviation.

Despite being thermo-stable, the tertiary structure of thanatin changed considerably during these three molecular dynamics (MD) simulations (Fig. 1B). The more we escalate temperature the more tertiary structure changed as the conformation of thanatin was denatured in water boiling temperature (Fig. 1B).

### In vitro thermal stability

The statistical analysis of the inhibition zone caused by the thanatin on *Escherichia* *coli* showed no significant difference between growth inhibition of unheated and heated thanatin until 50 min of heating at 100 °C (p < 0.05) (Table 2). The picture of the culture dish is presented in the Supplementary Information.

**Table 2:** The mean of diameter of inhibition zone (Millimeters) after heating the thanatin in 10, 30, and 50 min at 100 °C with standard error of the mean (SEM).

Bacteria strain	10 min	30 min	50 min	Unheated treatment	Negative control
*Escherichia coli* O157:H7	13.6 ± 0.33^a^	13.3 ± 0.33^a^	13.5 ± 0.33^a^	13 ± 0.33^a^	–

Negative control: The culture media without thanatin.

^a^No significant different between mean of diameter of inhibition zone.

### Protein modeling

**Figure 2:**
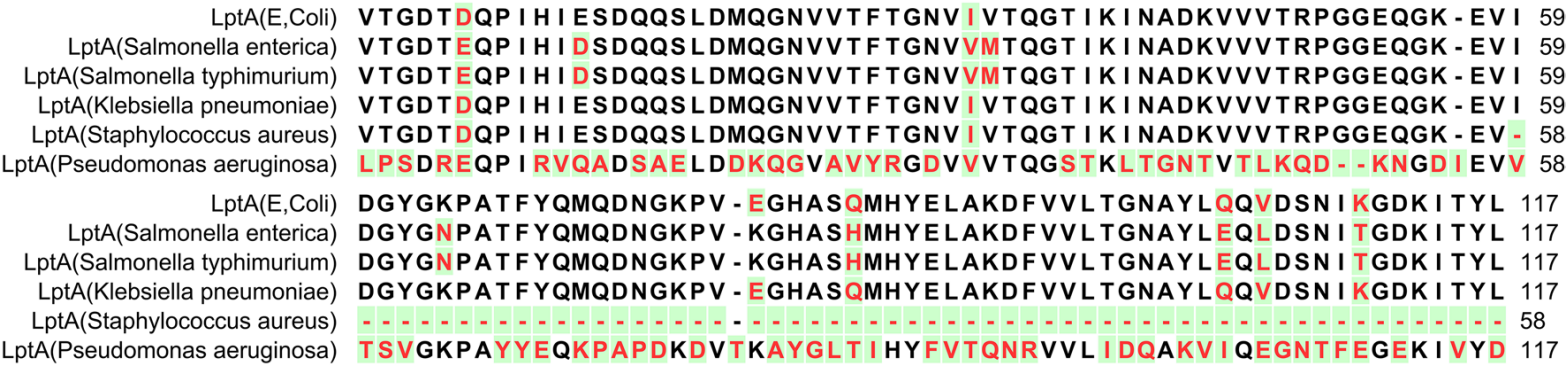
The alignment of LptA amino acid sequence of different pathogens from MIC test.

Apart from LptA amino acid sequence of *Pseudomonas aeruginosa*, the other sequences were almost identical (Fig. 2). The LptA amino acid sequence of *Staphylococcus aureus* (*S. aureus*) was not completely available. Finally, the LptA amino acid sequence of *Pseudomonas aeruginosa was* chosen for protein modeling.

The Ramachandran analysis before and after model refinement (Table 3) revealed that despite having low homology between the LptA sequence of *Pseudomonas aeruginosa* and the LptA sequence which retrieved from *Escherichia coli* (PDB entry: 6GDB) (~ 30%), swiss model could predict the tertiary structure of *Pseudomonas aeruginosa* LptA much better than the I-TASSER and Quark servers. The number of residues in most favored regions and residues in additional allowed regions for the model which was predicted by Swiss model was significantly higher than the similar data obtained from other servers. Hence, this model after refinement was considered for docking analysis.

**Table 3:** The Ramachandran analysis for predicted *Pseudomonas aeruginosa* LptA tertiary structures, before and after refinement, by three different procedures; Homology modeling, Threading, De novo (%).

The status of residues	After refinement			Before refinement		
	Swiss model	I-TASSER	Quark	Swiss model	I-TASSER	Quark
Residues in most favored regions	79.2	69.1	59.1	90.1	88.2	78.2
Residues in additional allowed regions	14.9	25.5	29.1	7.9	7.3	15.5
Residues in generously allowed regions	3.0	2.7	7.3	1.0	2.7	3.6
Residues in disallowed regions	3.0	2.7	4.5	1.0	1.8	2.7

### Docking analysis

**Figure 3:**
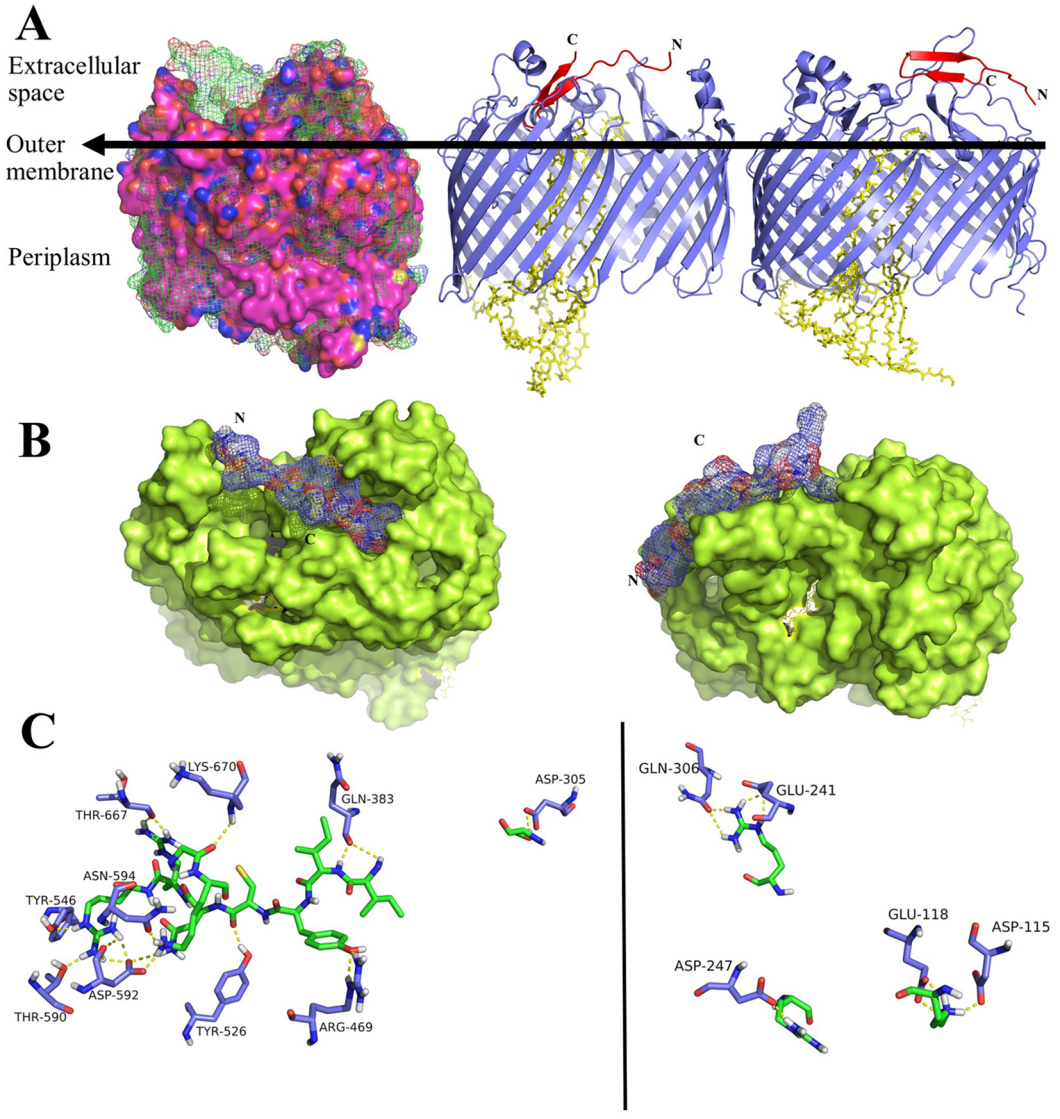
(**A**) Left: the alignment of crystallography structure of *Escherichia coli* LptD (surface mode, pink and blue) and *Pseudomonas aeruginosa* (mesh mode, green). Middle: the interaction of thanatin (red) with *Escherichia coli* LptD (blue)–LptE (yellow) complex. Right: the interaction of thanatin (red) with *Pseudomonas aeruginosa* LptD (blue)–LptE (yellow) complex. (**B**) Left: the head of outer membrane of *Escherichia coli* LptD (green) interacted with thanatin. Right: the head of outer membrane of *Pseudomonas aeruginosa* LptD (green) interacted with thanatin (purple blue). (**C**) (The residues of LptD are not labeled for more clarity) Left: the residue involvement in the interaction of thanatin (green) and *Escherichia coli* LptD (purple blue). Right: residue involvement in the interaction of thanatin (green) and *Pseudomonas aeruginosa* LptD (purple blue).

The conformation of LptD in *Pseudomonas aeruginosa* and *Escherichia coli* is significantly different and this fact leads to different site of interaction for thanatin. The differences between these two structures is more obvious at the outer membrane domain which is more occupied in *Pseudomonas aeruginosa* (Fig. 3A).

The docking results revealed that the loop of thanatin can penetrate to the outer membrane domain of LptD in *Escherichia coli* (Fig. 3A) while it could just interact with the exterior side section of LptD in *Pseudomonas aeruginosa* (Fig. 3B). According to the position of thanatin at the top of *Escherichia coli* LptD (Fig. 3B), it may prevent the transformation of LPS and other crucial components for biogenesis through LptA to LptDE path. On the other hand, thanatin at the exterior side section of *Pseudomonas aeruginosa* LptD would not disturb the transition of components.

The residue involvement analysis depicts that *Escherichia coli* LptD interact with thanatin more strongly (Fig. 3C). Just five residues of *Pseudomonas aeruginosa* LptD are engaged in the interaction of thanatin–LptD while this amount is considerably more for *Escherichia coli* LptD (Table 4). It can be seen that none of LptD residues which take part in this interaction, are identical between *Escherichia coli* and *Pseudomonas aeruginosa* (Table 4).

**Table 4:** The residue involvement of the interactions of LptA and LptD with thanatin in *Pseudomonas aeruginosa* and *Escherichia coli*, in addition to interaction distances (Å).

LptA (*Escherichia coli*)	Thanatin	Distance (Å)	LptA (*Pseudomonas aeruginosa*)	Thanatin	Distance (Å)	LptD (*Escherichia coli*)	Thanatin	Distance (Å)	LptD (*Pseudomonas aeruginosa*)	Thanatin	Distance (Å)
Ile36	Ile9	1.9	Asp54	Lys3	1.8	Asp305	Ser2	2.0	Asp115	Lys3	1.7
Ile38	Ile9	2.0	Asp54	Val6	1.9	Gln383	Ile8	2.8	Glu118	Lys3	1.8
Ile38	Cys11	1.7	Glu56	Ile8	2.3	Gln383	Ile9	2.0	Glu118	Lys3	1.8
Glu39	Arg13	1.8	Gln49	Ile9	2.0	Arg469	Tyr10	1.8	Glu241	Arg13	2.1
Ser40	Cys11	1.8	Tyr27	Ile9	2.1	Arg469	Tyr10	2.1	Glu241	Arg13	1.9
Ser40	Arg13	1.8	Asn52	Tyr10	1.9	Tyr526	Cys11	1.9	Gln306	Arg13	2.7
Asp41	Asn12	2.0	Glu17	Arg13	2.0	Asp592	Asn12	2.0	Gln306	Arg13	1.7
Gln43	Tyr10	2.4	Glu17	Arg13	1.9	Thr667	Arg13	1.6	Asp247	Arg20	1.7
Gln81	Arg20	2.6	Ser15	Arg13	1.8	Thr667	Arg13	1.7			
						Thr546	Arg14	1.9			
						Asp592	Arg14	2.0			
						Asp592	Arg14	2.3			
						Thr590	Arg14	1.6			
						Lys670	Gly16	2.5			
						Asn594	Lys17	1.6			
						Asp592	Lys17	1.7			

**Figure 4:**
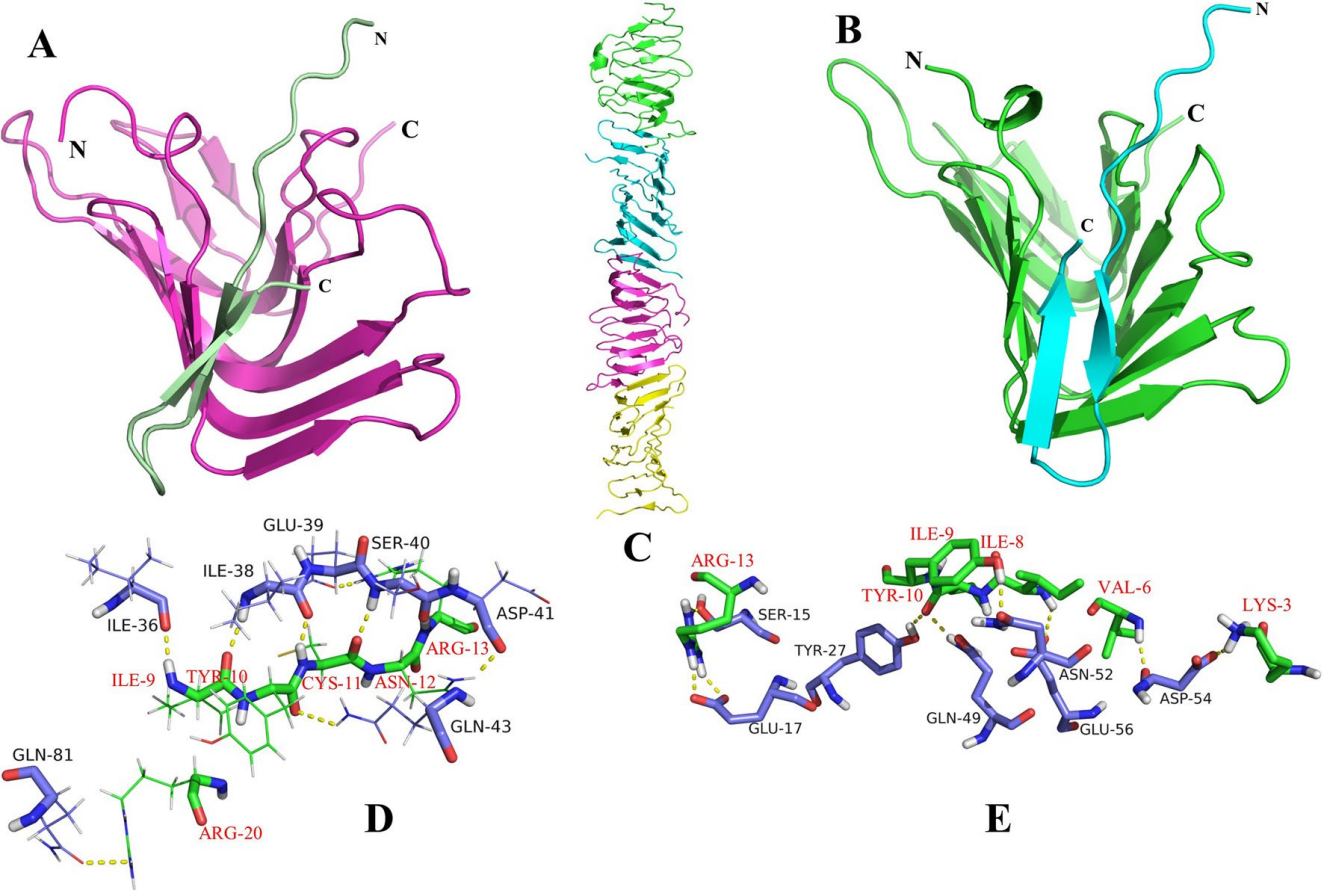
(**A,B**) the complex of thanatin (pale green and cyan)-LptA (hot pink and green) in *Escherichia coli* and *Pseudomonas aeruginosa* respectively. (**C**) The structure of LptA bridge in periplasm (PDB entry: 2R1A). (**D,E**) the residue involvement of thanatin (green)–LptA (purple blue) complex in *Escherichia coli* and *Pseudomonas aeruginosa*, respectively*.*

In contrary to the considerable different docking results of LptD and thanatin in *Pseudomonas aeruginosa* and *Escherichia coli,* the interaction of LptA and thanatin in these two pathogens revealed almost the same outcome (Fig. 4A,B). In this case, thanatin could cover the N-terminal of LptA in both of them and accordingly, the assembly of the LptA bridge (Fig. 4C) for transformation of LPS and other necessary components for biogenesis of bacteria would be disturbed. Apart from the approximately similar position of interaction for thanatin in LptA of *Pseudomonas aeruginosa* and *Escherichia coli,* the residue involvement analysis of docking revealed that both interactions involve the same number but different residues (Fig. 4D,E).

## Discussion

Motivations for antibiotic use in animal industries fall into three categories: growth promotion, disease prevention and treatment. Subsequently the number of resistant bacteria was expanded as big challenges in many developing countries^20^. On the other hand, antibiotic resistance issue has faced animal industry with enormous problems and making lots of problems on these industries such as economic losses, unhealthy effects on human through transfer of antibiotics resistant bacteria^2^. The use of antibiotic alternative such as AMPs is essential program^21^. The fact that AMPs are effective against a wide spectrum of microorganism, for instance multidrug resistance pathogens, Gram-positive and negative bacteria^22^. There are different types of AMPs with various functions for antibacterial activity might cause of drug resistant in some of bacteria similar antibiotics^11^. This action depended on the properties of the lipid membrane of bacteria or the peptide’s structure. Documents showed that in addition to acting of AMPs on cell membrane, there are several other antibacterial mechanisms for example inhibiting protein folding, enzyme or intracellularly activity^23^. Thanatin (GSKKPVPIIYCNRRTGKCQRM) is one of the members of cationic AMPs family and isolated from insect after immune challenge. It can inhibit the growth of some antibiotic resistant bacteria for instance *Escherichia coli*^5, 7, 8^ also, inhibits the growth and biofilm formation of methicillin-resistant *S. epidermidis* (MRSE) in vitro and in vivo conditions^10^.

Although pore-forming AMPs disrupt inner membrane of bacteria, agglutination peptide target outer membrane components of bacteria cell such as thanatin. These AMPs plays a pivotal role for destroying bacteria without permeabilize the cell membrane^24, 25^.

Mechanism of cell agglutinating AMPs is poorly understood. Sinha et al. (2017) showed the effect of thanatin on Gram-negative bacteria. They assumed that thanatin in complex with LPS has a four-stranded antiparallel β-sheet in a ‘head–tail’ dimeric topology while in free solution, it forms an antiparallel β-hairpin conformation and this structure has higher hydrophobicity and cationicity in interactions with sites of LPS. This structure of thanatin able binding two or more LPS molecules at the distal ends and permit bacterial cells vicinity. Sinha et al.^14^ also claimed that disorder on LPS-outer membrane integrity and cell-surface charge neutralization after being treated with thanatin may perhaps facilitate cell–cell aggregation and agglutination^14^. It seems thanatin could be a desirable alternative candidate instead of common antibiotics^26^. In addition, several newly proved modes of action have been proposed for the antimicrobial activity of thanatin^27, 28^. Ma et al.^28^ have claimed that thanatin is able to inhibit the enzymatic activity of New Delhi metallo-β-lactamase-1 (NDM-1) by displacing zinc ions from the active site, and reverses carbapenem resistance in NDM-1-producing bacteria. By hydrolyzing almost all clinically used β-lactam antibiotics, NDM-1 positive pathogens are a reason of global concern for antimicrobial resistance. In addition, thanatin can disrupt the outer membrane of NDM-1-producing bacteria by competitively displacing divalent cations on the outer membrane and inducing the release of lipopolysaccharides. But this result only belong to the NDM-1-producing pathogens and non NDM-1-producing pathogens needs to be eliminated by other pathways^28^. Vetterli et al.^27^ have proposed a broader procedure. They depict that insect-derived antimicrobial peptide, thanatin, targets LptA and LptD in the network of periplasmic protein–protein interactions which is required to assemble the Lpt complex. The Lpt complex (lptA to LptG) are the proteins which are necessary for biogenesis of the asymmetric Gram-negative bacterial OM^27^. Accordingly, the inactivation of this path leads to the inhibition of LPS transmission and OM biogenesis. In contrary to the Ma et al. findings, this mode of action covers all Gram-negative pathogens, not only NDM-1 positive bacteria^28^.

The MIC and MBC antibacterial methods showed antimicrobial activity against all bacteria except against *Klebsiella pneumoniae* and *Pseudomonas aeruginosa* (from poultry disease). It might be due to different mode of action of thanatin or maybe higher concentrations of thanatin were needed to kill these bacteria. According to the Vetterli et al. (2018) findings, different MIC results for thanatin against various pathogens raised a question. Could these differences be originated from the different quality (regarding to the number and distance of interactions which leads to higher binding energy) of interactions between thanatin and LptA and/or LptD of pathogens? To answer this question, we used two different pathogens with significant different MIC results in our research which confirmed previous investigations^7^. In this study, the activity of thanatin against *Escherichia coli* was much more than *Pseudomonas aeruginosa.* Therefore, we obtained the tertiary structure of LptA protein of *Pseudomonas aeruginosa* through protein modeling. Afterward, we used the *Escherichia coli* NMR structure of LptA (PDB entry: 6GD5), LptD (PDB entry: 4RHB), and also LptD crystallography structure of *Pseudomonas aeruginosa* (PDB entry: 5IVA) for docking studies.

The docking site of LptA of *Escherichia coli* and *Pseudomonas aeruginosa* were identical while thanatin positioned in a significantly different location of LptD of *Escherichia coli* in comparison with *Pseudomonas aeruginosa.* In a glance, it can be inferenced that the outer membrane of LptD which is the interaction site of this protein with thanatin^27^ is more condense in *Pseudomonas aeruginosa* (Fig. 3A)^29^ in comparison with the same position of LptD in *Escherichia coli*^30^. In this case, Leu239-Arg265, Ser524-Arg530, and Asp596-Gln602 amino acid sequences of LptD prevent the thanatin to penetrate to the outer membrane layer of LptD of *Pseudomonas aeruginosa* (Fig. 3B). On the other hand, thanatin could locate into the outer membrane layer of *Escherichia coli.* It is likely that this position of thanatin prevents the transmission of LPS in *Escherichia coli* while thanatin would not be an obstacle for this action in *Pseudomonas aeruginosa*. As a result, thanatin can defect the membrane biogenesis of *Escherichia coli* more efficiently in comparison with *Pseudomonas aeruginosa* because it can prevent the LPS transmission more efficiently by blocking the LptD.

In summary, the RMSD of thanatin in different thermal conditions revealed thermal stability of thanatin in normal body temperature of dairy cattle and avian but in water boiling temperature at sea level, despite of being almost stable, thanatin may lose its native structure which can affect its function and defiantly, this temperature would not be considered as a biologically important temperature but maybe important in food and drug development^31^. According to native structure of thanatin in dairy cattle and avian normal body temperatures, it can maintain its functionality and based on MIC test, it can eliminate various pathogens on these temperatures.

The current study investigated the effects of thanatin on some poultry and farm animal pathogens. Almost all of these bacterial species were treated by thanatin and were killed. Furthermore, resistance of thanatin against changes in different temperatures indicates its stability against heat. This is very important feature for peptide as drug. Our study results support the note that the thanatin peptide may candidate as naturel alternative instead common antibiotics for animal. In addition, we have shown that the level of activity of thanatin might be affected by the quality of interaction of LptA/D–thanatin.

## Methods

### Recombinant thanatin

This peptide was utilized from our previous study^12^.

### Bacterial strains

The *Escherichia coli, Escherichia coli k99, Salmonella typhimurium, Salmonella Enteritidis* (isolated from poultry diarrhea), *Staphylococcus aureus, Klebsiella pneumoniae, Pseudomonas aeruginosa and Salmonella Enteritidis* (isolated from poultry intestine infection) as well as *Klebsiella pneumoniae, Salmonella Enteritidis, Staphylococcus aureus* (isolated from cattle mastitis), *Escherichia coli* O157:H7 (isolated from calf diarrhea) were obtained from Mashhad School of Veterinary Medicine Collection, Ferdowsi University of Mashhad, Iran.

### Analysis with SDS-PAGE

The SDS-PAGE used for monitoring of peptide that secreted in culture media. 30 µL of the culture media supernatant (transfected cells by recombinant and self-ligated pcNDA3.1+) were run on SDS-PAGE in Tris/Glycine/SDS buffer using a 16% polyacrylamide gel and was stained with Coomassie Brilliant Blue^12^. This peptide was not purified, thus the concentration of the peptide was estimated with the GelQuant software v 1.8.2 (www.biochemlabsolutions.com) from the SDS-PAGE image.

### Minimum inhibitory concentration (MIC) and minimum bactericidal concentration (MBC)

The MIC was used based on a microbroth dilution method in a 96-well microtiter with four replications. The bacteria were cultured overnight on Mueller–Hinton broth and the optical density (OD _625 nm_) was adjusted equal to 0.08–0.1. Then distributed in 100 μL volumes into a 96-well microtiter plate. A 100 μL medium containing thanatin peptide from stock solution was used to prepare a serial dilution (3.125, 6.25, 12.5, 25, 50, 100 and 200 μg/mL) and 100 μL from each dilution was added to each 96-well microtiter plates. The plate was incubated at 37 °C for 24 h without shaking. In this study, the lowest concentration of thanatin that could inhibit the bacterial growth was used as MIC for different bacterial isolates^12^. The McFarland standard 0.5 was used as standard to measure the turbidity. Medium without inoculum was considered as negative control and Gentamicin (μg/mL) used as positive control^32^. After MIC test, 100 μL of dilutions with no bacterial growth pour-plated on Mueller–Hinton agar medium and incubated at 37 °C for 24 h. The MBC was determined based on the lowest bactericidal concentration showing no visible growth on culture plates^32, 33^.

### In silico thermal stability

In order to predict the thermal-stability of thanatin under different time and thermal circumstances, MD simulations was used. Three different temperatures were considered according to the origin of pathogens in MIC test. Accordingly, normal dairy cattle body temperature (312 K)^34^, normal avian body temperature (315 K)^33^ and water boiling temperature at sea level were used.

### In vitro thermal stability

Thermal stability was performed by heated thanatin at 100 °C for 10, 30, and 50 min and its antibacterial activity on *Escherichia coli* O157:H7 was investigated. The culture was at 37 °C for 24 h and in Mueller–Hinton agar medium. The culture media (DMEM) without thanatin was considered as negative control group. This experiment was conducted in a completely randomized design with three replications. Data were subjected to a one-way ANOVA analysis by the SAS software, Version 9 for the SAS System for Windows (SAS is a registered trademark of SAS Institute Inc., Cary, NC, USA).

### Dynamic simulation

In order to predict the thermal-stability of thanatin, the crystallography structure of this peptide (PDB entry: 8TFV) was used as an input. MD simulation performed through GROMACS 5.0.1^35^ using GROMOS 54a7^36^ force field, SPC/E water molecules^37^, and cubic box with periodic boundary conditions. Particle Mesh Ewald (PME) summation method was used to calculate the total electrostatic energy in each periodic box the^37^. The other non-bonded interactions were calculated by L–J model with 10 Å cutoff distance. The steepest-descent algorithm was applied for energy minimization. In order to fix the chemical bonds among atoms of the protein, LINCS algorithm^36^ was used and similarly, SETTLE algorithm^38^ was applied for solvent molecules. To retain 312 K, 315 K, and 373 K as dairy cattle normal body temperature, avian normal body temperature, and water boiling temperature at sea level, respectively, and to maintain pressure of each system during MD simulations, pressure and temperature baths were applied using the Berendsen coupling algorithm^38^. Weak-coupling algorithm was used for the temperature and pressure regulation with a coupling time of 1.0 ps. 100 ns and 200 ns MD simulation were performed for normal body temperatures and water boiling temperature at sea level, respectively. The root-mean-square deviation (RMSD) of thanatin α-carbons was plotted versus time during the 100 and 200 ns MD simulation.

### Protein modeling

According to the pathogens of MIC test, the LptA amino acid sequence of *Staphylococcus aureus (WP_000293604.1*), *Escherichia coli *(WP_024182920.1), *Klebsiella pneumoniae* (CDK70720.1), *Salmonella Enteritidis* (ADX19108.1)*, Pseudomonas aeruginosa* (WP_034043186.1), *and Salmonella Typhimurium* (ADX19108.1) were retrieved from National Center for Biotechnology Information (NCBI) database. To investigate the differences of amino acid sequence of LptA for the mentioned pathogens, the alignment of these sequences was done through CLC sequence viewer 8.0 (https://www.qiagenbioinformatics.com/). Afterwards, based on the MIC test results and differences in amino acid sequence of LptA, the most sensitive (*Escherichia coli*) and one of the most resistance (*Pseudomonas aeruginosa*) pathogens against Thanatin were chosen for further analysis to inquire the relationship between LptA and the functionality of Thanatin. Accordingly, the LptA amino acid sequence of *Pseudomonas aeruginosa* was considered for protein modeling. The functionality of thanatin against *Pseudomonas aeruginosa* is less than *Escherichia coli* and this fact might be due to the different pattern of interaction of thanatin with LptA and LptD proteins. The crystallography structure of LptD of *Pseudomonas aeruginosa* (PDB entry: 5IVA) *and Escherichia coli (*PDB entry: 4RHB) and also LptA of *Escherichia coli* (PDB entry: 6GDB) is available while there is no tertiary structure information of LptA of *Pseudomonas aeruginosa.*

Prediction of tertiary structures was done by three different procedures and algorithms. Swiss model^39^, I-TASSER^40–42^, and Quark^43, 44^ servers were utilized for homology modeling, threading, and de novo modeling, respectively. Using various algorithms was due to low homology between LptA of *Escherichia coli* and *Pseudomonas aeruginosa* (~ 30%).

The quality of models was improved by Refold server^45, 46^. According to the Ramachandran analysis of residues, the best model was chosen for further steps.

### Docking analysis

The interaction of thanatin and N-terminal beta strands of LptD was previously identified were the connection of LptD and LptA will be disrupted through thanatin. In this study, we investigated the interaction of thanatin and the outer membrane domain of LptD in *Escherichia coli* and *Pseudomonas aeruginosa* by Cluspro 2.0^47–49^ to see if thanatin could act as an obstacle for the entry of vital components for biogenesis and find new sites of interaction. In this regard the periplasmic sections of LptD for *Escherichia coli* (*PDB entry:4RHB*) and *Pseudomonas aeruginosa* (*PDB entry:5IVA*) were masked to inquire new^50^. The PDB structure of thanatin was extracted from *Escherichia coli* LptA-Thanatin complex (PDB entry: 6GD5) by Pymol software (The PyMOL Molecular Graphics System, Version 1.8, Schrödinger, LLC)^51^. The best model of docking was chosen based on a balance between electrostatic-favored, hydrophobic-favored, and van-der-waals + Electrostatic.

On the other hand, the best predicted and refined model for LptA of *Pseudomonas aeruginosa* was used for thanatin-LptA docking analysis. Similarly, the C-terminal half of LptA which is not the interaction site of LptA^27^ was masked^50^ and the result was compared to the NMR complex of LptA-thanatin in *Escherichia coli* (PDB entry:6GD5).

The residue involvement and the length of bonds for dockings were analyzed and compared for *Escherichia coli* and *Pseudomonas aeruginosa*. The result of thanatin-LptA for *Pseudomonas aeruginosa* was compared with the NMR structure of *Escherichia coli* LptA-Thanatin complex (PDB entry: 6GD5).

## Supplementary Information

Supplementary Information.
