# Supplementary material for: Antibacterial effects assessment on some livestock pathogens, thermal stability and proposing a probable reason for different levels of activity of thanatin
Source: Sci Rep. 2021 May 25;11:10890. doi: 10.1038/s41598-021-90313-4 (PMC8149819; doi:10.1038/s41598-021-90313-4)
Supplement: Supplementary file 1 — Supplementary Information. [file 41598_2021_90313_MOESM1_ESM.docx]

**Antibacterial effects assessment on some livestock pathogens, thermal stability and proposing a probable reason for different levels of activity of thanatin**

Ali Javadmanesh^*1,2^, Elyas Mohammadi^1^, Zahra Mousavi^1^, Marjan Azghandi^1^, Abass Tanhaiean^3^

^1^ Department of Animal Science, Faculty of Agriculture, Ferdowsi University of Mashhad, Mashhad, Iran.

^2^ Stem Cell Biology and Regenerative Medicine Research Group, Research Institute of Biotechnology, Ferdowsi University of Mashhad, Mashhad, Iran.

^3^ Department of Plant Breeding, Faculty of Agriculture, Shahrood University of Technology, Shahrood, Iran.

Correspondence: Dr. Ali Javadmanesh, Department of Animal Science, Faculty of Agriculture, Ferdowsi University of Mashhad, Mashhad, Iran. Postal Code 9177948974.

E-mail: javadmanesh@um.ac.ir


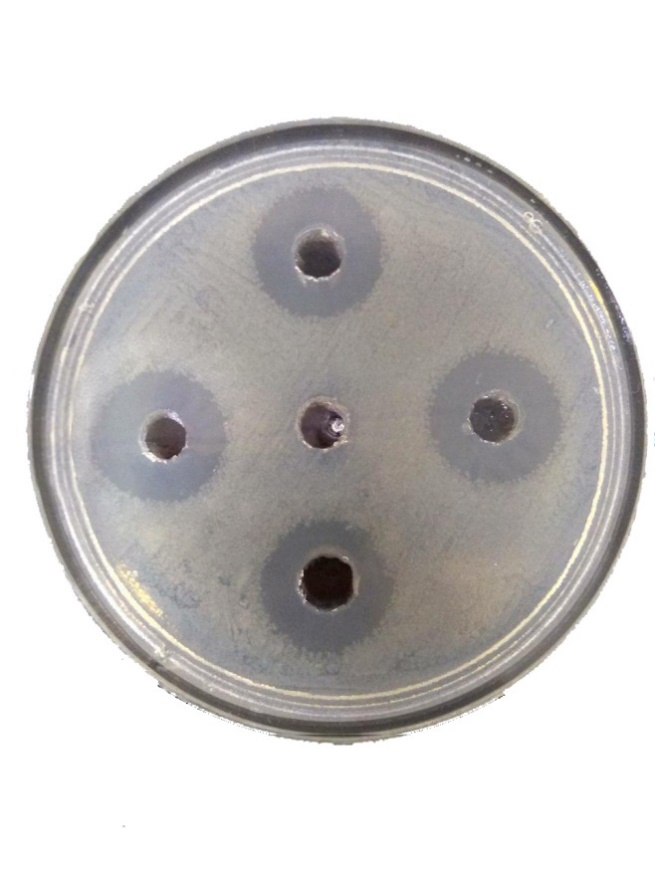


4

1

3

2

5

Supplementary figure: The effect of heating (100 ^°C^) on antibacterial activity of thanatin peptide against *E. coli* 0157 H7 by well-diffusion method. **1, 2, 3:** Duration of heating peptide at 10,30 and 50 minutes, respectively. **4:** Unheated thanatin. **5:** The media culture without thanatin.
